# Changing mechanical properties of photopolymerized, dityrosine-crosslinked protein-based hydrogels

**DOI:** 10.3389/fbioe.2022.1006438

**Published:** 2022-09-12

**Authors:** Sandra Haas, Saskia Körner, Laura Zintel, Jürgen Hubbuch

**Affiliations:** Institute of Process Engineering in Life Sciences, Section IV: Molecular Separation Engineering, Karlsruhe Institute of Technology (KIT), Karlsruhe, Germany

**Keywords:** protein-based hydrogels, visible-light induced photopolymerization, urea, protein unfolding, BSA—bovine serum albumin, casein

## Abstract

Hydrogels based on renewable resources are a promising class of materials for future applications in pharmaceutics, drug delivery and personalized medicine. Thus, optional adjustments of mechanical properties such as swelling behavior, elasticity and network strength are desired. In this context, hydrogels based on the biological raw materials bovine serum albumin and casein were prepared by dityrosine-crosslinking of their tyrosine residues through visible light-induced photopolymerization. Changing the tyrosine accessibility by urea addition before photopolymerization increased the storage modulus of the hydrogels by 650% while simultaneously being more elastic. Furthermore, contributions of the buffer system composition, variation of protein concentration and storage medium towards mechanical properties of the hydrogel such as storage moduli, elasticity, fracture strain, compressive strength and relative weight swelling ratio are discussed. It could be shown, that changes in precursor solution and storage medium characteristics are crucial parameters towards tuning the mechanical properties of protein-based hydrogels.

## 1 Introduction

Hydrogels are three-dimensional (3D) polymer networks with the ability to expand their volume in aqueous solutions (Alemán et al., 2007). Since the first hydrogel formulation was published, the number of publications on hydrogel formulations and applications in the field of pharmaceutics and medicine steadily grew over the years (Wichterle and Lím, 1960; Elzoghby et al., 2011; Van Vlierberghe et al., 2011; Ullah et al., 2015). In this context, renewable and naturally occurring resources like proteins and peptides, which show high biocompatibility and biodegradability, represent potential raw material sources for hydrogel formulations (Jonker et al., 2012; Abaee et al., 2017).

Hydrogel formulations can be classified by different properties, such as the crosslinking mechanism, physical properties, external stimuli behavior, and monomer or polymer chain source (Khan et al., 2016). Protein- or peptide-based hydrogels can either self-aggregate after denaturation by heat, salt-, urea- or acid-induced gelation as described for hen egg white lysozyme (Yan et al., 2006), soy proteins (Maltais et al., 2009; Chien et al., 2014), and whey proteins including β-lactoglobulin (Remondetto et al., 2002; Gosal et al., 2004; Akkermans et al., 2008) and bovine serum albumin (BSA) (Lu et al., 2020). Other than those, enzymatic crosslinking or chemical crosslinkers - with the drawback of the need of added functional groups for crosslinking or the use of potentially toxic initiators - are commonly applied to obtain hydrogels (Totosaus et al., 2002; Elzoghby et al., 2011). Recently, the potential of visible-light induced hydrogelation mediated by tris(2,2′-bipyridyl)dichlororuthenium (II) (Ru (bpy)_3_Cl_2_) in the development of 3D printable bio-based materials was shown (Sakai et al., 2018). Thereby, phenolic hydroxy groups–which are naturally present in tyrosine residues of proteins–are crosslinked to dityrosine finally inducing gelation (Fancy and Kodadek, 1999; Fancy et al., 2000).

Dityrosine crosslinks are found to be contributing to the elastic properties of natural materials such as resilin and elastin (Andersen, 1964; Partlow et al., 2016). So far, studies focus on dityrosine crosslinking of different peptide sequences include elastin-like proteins (Elvin et al., 2005a; Ding et al., 2013; Jeon et al., 2015; Camp et al., 2020), mussel adhesive proteins (Jeon et al., 2015), unmodified proteins (e.g., gelatin, fibrinogen, and maltose binding protein) (Elvin et al., 2010; Hughes et al., 2020) and unmodified proteins at different folding states (I27, Protein L, BSA, maltose binding protein) (Da Silva et al., 2017; Khoury et al., 2018; Hughes et al., 2022). Recently, the influence of the reaction rate towards the viscoelasticity of folded protein hydrogels was further assessed (Aufderhorst-Roberts et al., 2020). However, there is a lack of knowledge on the influence of the type of protein and reaction conditions. Likewise, processing parameters, such as the formulation buffer and storage medium impact mechanical properties and storability of hydrogels before the actual application.

In this manuscript, we assessed dityrosine-crosslinked hydrogels—crosslinked by ruthenium-mediated photopolymerization—derived from two naturally occurring proteins, the globular BSA and a conjugated casein. To allow tailor-made development of intelligent materials, key processing parameters such as buffer composition and characteristics during precursor solution preparation, the presence of urea and protein concentration as well as different storage media were described towards their influence on mechanical properties.

## 2 Materials and methods

### 2.1 Precursor solution formulation

#### 2.1.1 Buffer stock solution

If not stated otherwise, all chemicals were purchased from Merck KGaA (Darmstadt, DE). 20 mM sodium phosphate buffer (SPB) or a 25 mM multi-component buffer (MCB) containing different urea concentrations (0–4 M) were prepared with ultrapure water (PURELAB Ultra, ELGA LabWater, Lane End, United Kingdom), while Gibco^®^ Dulbecco’s phosphate-buffered saline (DPBS, Life Technologies Corporation, Grand Island, US-NY) was used as purchased. The MCB had a global capacity of 25 mM in the range of pH 6 to pH 9 and consisted of 47 mM N-[tris(hydroxymethyl)methyl]-3-aminopropanesulfonic acid (TAPS), 11 mM 3-morpholino-2-hydroxypropanesulfonic acid (MOPSO) and 38 mM sodium citrate. At a buffer temperature of 22°C, the buffer was pH-adjusted using 4 M sodium hydroxide solution and filtered through a 0.45 µm cellulose acetate membrane (Pall Corporation, New York, NY, United States).

#### 2.1.2 Protein stock solutions

Protein stock solutions of bovine serum albumin (BSA) and casein (EMD Millipore Corporation, Billerica, MA, United States) were prepared with a concentration of 120 mg/ml in the respective buffer solution using a dual asymmetric centrifuge (DAC) at 2,500 rpm (SpeedMixer^®^ DAC 150.1 FVZ-K, Hauschild GmbH & Co., KG, Hamm, DE). Protein concentrations were determined with a NanoDrop 2000c UV-Vis spectrophotometer (Thermo Fischer Scientific, Waltham, MA, United States) using the extinction coefficients ε_BSA,280nm_ = 0.67 L/(g * cm) (Amrhein et al., 2015) and ε_Casein,280nm_ = 0.73 L/(g * cm) (experimentally determined).

Protein purification to reduce impurities (e.g., production buffer salts) was either performed by dialysis or ultrafiltration. Dialysis of protein stock solution was performed using SnakeSkin™ dialysis tubing (Thermo Fisher Scientific, Waltham, MA, United States) with a 10 kDa molecular weight cut off (MWCO) and 100-fold buffer excess. Two buffer exchanges were performed, the first after >2 h, the second after > 2 more hours. Alternatively, Vivaspin^®^ ultrafiltration units (Sartorius Stedim Biotech, Göttingen, DE) were used according to the manufacturer’s recommendation for protein purification or subsequent dialysis to reach higher stock solution concentrations (MWCO_BSA_ = 30 kDa; MWCO_Casein_ = 10 kDa).

#### 2.1.3 Photoinitiator and co-factor

The photoinitiator tris(2,2′-bipyridyl)dichlororuthenium (II) hexahydrate (Ru(bpy)_3_Cl_2_6H_2_O) was diluted in the corresponding buffer to a concentration of 5 mM and stored at 4°C. The co-factor ammonium persulfate [APS, chemical formula: (NH_4_)_2_S_2_O_8_] was diluted with a concentration of 2 M in the corresponding buffer, stored as aliquots at −20°C and thawed directly prior to usage.

#### 2.1.4 Precursor solution

**FIGURE 1:**
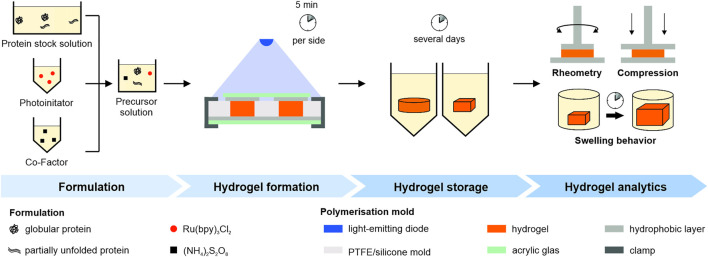
Schematic overview of the experimental workflow. Pre-processed protein, photoinitiator and co-factor stock solution were mixed to generate a precursor solution. Polymerization was achieved through illumination in a mold. Hydrogels were stored under different conditions and mechanical properties were determined either by oscillatory rheometry, swelling studies or uniaxial compression tests.

Formulation buffer, protein and photoinitiator stock solutions were mixed using the DAC (2,500 rpm, 5 min). Afterwards, APS stock solution was added and mixed in a light-protected container (2,500 rpm, 2 min) to generate a uncrosslinked precursor solution (Figure 1 *Formulation*). The concentration of Ru(bpy)_3_Cl_2_ (0.25 mM) and APS (100 mM) was kept constant, while protein source, protein concentration and formulation buffer composition were varied (Supplementary Table S1).

### 2.2 Hydrogel formation

The precursor solution was transferred into a dedicated mold (Figure 1 *Hydrogel formation*). We used the following molds: A cylindrical polytetrafluoroethylene (PTFE) mold (diameter 10 mm, height 3 mm), a cylindrical silicone mold (diameter 12.5 mm, height 3 mm) and a cuboidal silicone mold (side lengths 5 mm, height 3 mm). The mold was covered on top and bottom with transparent acrylic glass and a hydrophobic layer in between and irradiated for 5 min from atop and below using a blue emitter at 457 nm (LZ4-00B208, LED Engin Inc., San Jose, CA, United States) with a radiant flux of 3.9 W in a distance of 7 cm.

### 2.3 Storage and analytics

#### 2.3.1 Hydrogel storage

Before mechanical characterization, polymerized hydrogels were stored under different storage conditions as stated in Supplementary Table S1 in the supplementary information. Subsequently, rheometric analysis or uniaxial compression analysis were performed as analytical methods.

#### 2.3.2 Oscillatory frequency sweeps

Oscillatory measurements were performed with hydrogel discs at 22°C on a Physica MCR 301 plate rheometer (Anton Paar GmbH, Graz, AT) equipped with a plate-plate geometry (10 mm diameter). The linear viscoelastic region (LVR) was determined using amplitude sweeps for angular frequencies *ω* = 1 and 25 rad s^−1^ and shear stress *τ* between 5 and 10.000 Pa (*n* = 2). Frequency sweeps were performed within the LVR using *τ* = 10 Pa and ω = 1–25 rad s^−1^ (*n* = 3).

#### 2.3.3 Uniaxial compression analysis

Uniaxial compression tests were performed with hydrogel discs on a universal testing machine (zwickiLine Z0.5TN, ZwickRoell GmbH & Co., KG, Ulm, DE) equipped with a load cell Xforce HP 100 N and stainless-steel compression platens (30 mm diameter) at a uniform velocity of 2 mm/min until sample breakdown after a pre-force of 0.2 N was reached (*n* = 3). Two parameters were determined, the engineered stress *σ* = F/A_0,_ where F is the force applied and A_0_ is the original cross-sectional area and the engineered strain *ε* = (L−L_0_)/L_0,_ where L is the sample length and L_0_ is the original sample length at the applied pre-force. The fracture strain ε_max_ refers to the engineered strain at sample fracture. Accordingly, the compressive strength σ_max_ refers to the engineered stress at sample fracture.

#### 2.3.4 Swelling studies

Swelling studies were performed with hydrogel cuboids which were stored in DPBS and MCB with the production pH without urea. The cuboids were weighed directly after polymerization and subsequently after 1, 2, 3, 7, 10, and 14 days. The relative weight swelling ratio m_rel_ of the hydrogels was calculated as m_rel_ = m/m_o_, where m and m_o_ are the masses of the hydrogel specimen at time points *t* and *t*_*0*_ respectively (*n* = 3). The storage medium was renewed after each measurement up to a final 60-fold buffer excess compared to the hydrogel volume.

#### 2.3.5 Statistical analysis

All experiments were performed in triplicates and data have been given as mean ± standard deviation. Statistical analysis for oscillatory frequency sweeps was performed using the two-sided Wilcoxon ranksum test with a *p*-value below 0.05 being classified as statistically significant and marked with a single asterisk (*). Statistical analysis for uniaxial compression tests were compared using a paired Student’s *t*-test with *p*-values less than 0.05 being considered significant and marked with a single asterisk (*). Beforehand, distribution normality was assessed with a Shapiro-Wilk test with a maximum significance level *α* = 0.05.

## 3 Results and discussion

### 3.1 Buffer components

In order to evaluate potential factors influencing the mechanical properties of protein-based hydrogels, varying formulation buffer compositions, proteins and protein concentrations as well as storage condition were examined. Initially, hydrogels prepared by visible light-induced chemical crosslinking of 100 mg/ml BSA were polymerized in a sodium phosphate buffer (SPB) at pH 8 containing up to 3 M urea. When using the SPB at pH 8, the generation of the required protein stock solutions containing higher urea concentrations, was prevented by urea-induced gelation (Totosaus et al., 2002). The influence of buffer pH was analyzed subsequently, applying a multi-component buffer system (MCB), which was used for all further experiments. Buffer components known to stabilize the native structure of BSA - here MOPSO and TAPS (Taha et al., 2011; Gupta et al., 2013) - enabled the preparation of 100 mg/ml BSA gels at pH 7 and pH 8 and urea concentrations up to 4 M. The third buffer component, sodium citrate is known to increase the solubility of casein (Haller and Pallansch, 1960; Udabage et al., 2000) which was used as a second protein.

### 3.2 Network density of bovine serum albumin-based hydrogels

**FIGURE 2:**
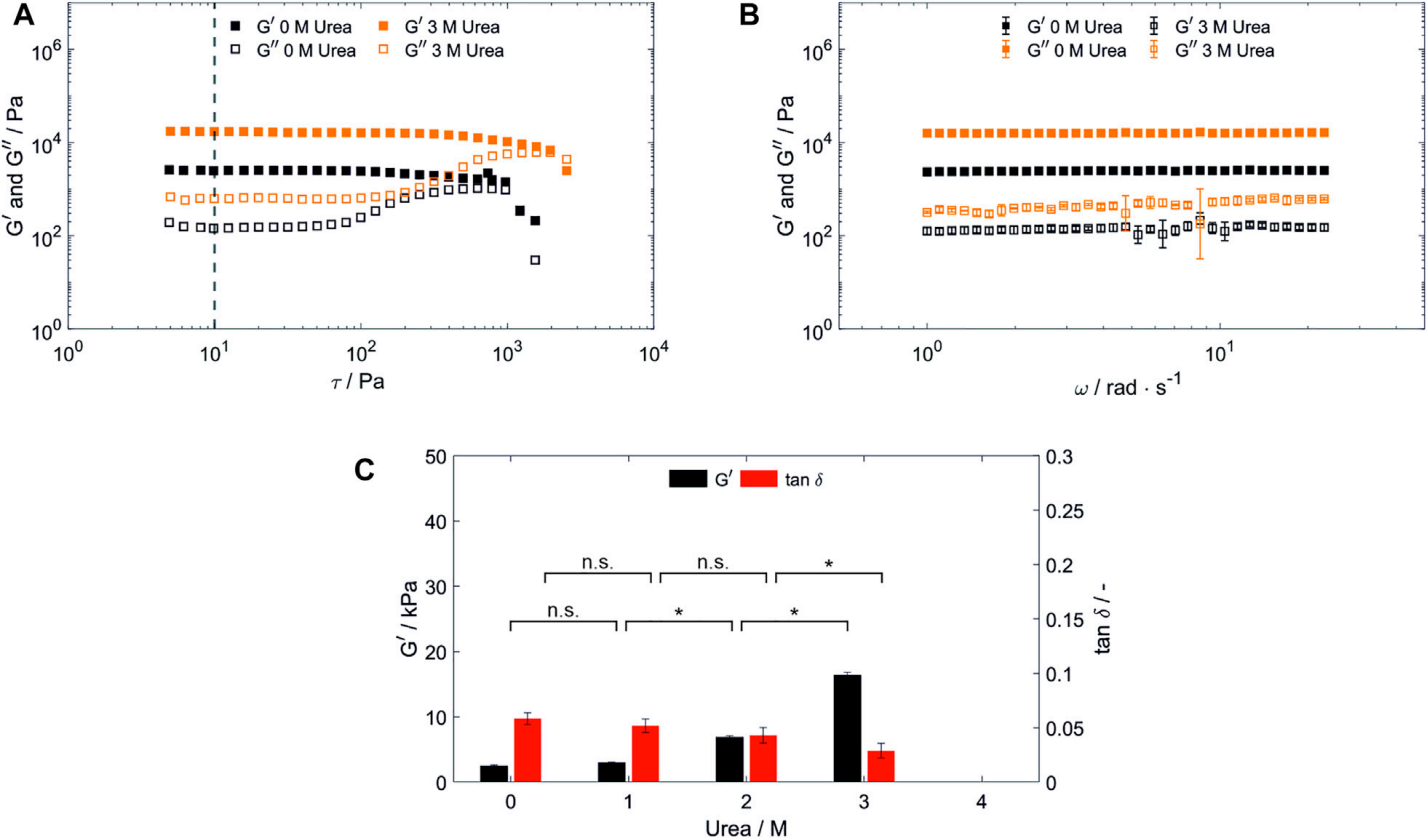
Characterization of hydrogels containing 100 mg/ml bovine serum albumin prepared in SPB. **(A)** Shear stress dependent (*ω* = 25 rad s^−1^, 22°C) and **(B)** frequency-dependent [*τ* = 10 Pa, highlighted by a vertical line in **(A)**, 22°C] oscillatory shear test of hydrogels prepared and stored in a 20 mM SPB at pH 8 without urea and with 3 M urea present in the precursor solution and storage medium. (*n* = 3) **(C)** Hydrogel storage modulus (G’) and loss factor (tan δ) in dependency of the urea content in the precursor solution and storage medium. (*n* = 3) Abbreviations: n.s, not significant, *p < 0.05.

Network failure of polymerized BSA-based hydrogels was analyzed using stress-dependent oscillatory rheology. It is indicated by an increasing loss modulus (G”) until a sudden breakdown of the storage modulus (G’) during amplitude sweeps (Figure 2A). Network failure at strains above 88 Pa was observable as indicated by the linear viscoelastic region (LVR), whereby the hydrogel network prepared with 2 and 3 M urea present in the SPB were more stable compared to the one prepared without urea (Supplementary Information S2).

G’, which is used as a measure for the network density, was determined by frequency sweeps. These were carried out at a constant shear stress of 10 Pa, being in the LVR for all hydrogels discussed in this manuscript. For all hydrogels tested, G’ dominates G” across the whole frequency range applied (1–25 rad s^−1^) confirming gel-like behavior (Almdal et al., 1993) as exemplarily depicted in Figure 2B. For BSA-based hydrogels prepared in SPB, a significant increase in G’ for increasing urea concentrations occurred for samples containing more than 1 M urea (Figure 2C). In numbers, G’ was increased by 650% from 2.47 ± 0.18 kPa of the 0 M urea samples to 16.04 ± 0.72 kPa for 3 M urea, while G” was increased by 313% from 0.14 ± 0.03 kPa to 0.45 ± 0.14 kPa.

The addition of urea disrupts intramolecular hydrophobic interactions and the protein unfolds (partially). Intermolecular interaction, such as those leading to urea-induced gelation, therefore may increase, resulting in a higher network strength. In addition, due to their amphiphilic nature, tyrosines can be located both, on the protein surface or inaccessibly in the hydrophobic core of native proteins (Joshi et al., 2004). Thus, solvent accessibility for the tyrosine residues is altered upon addition of urea (Nnyigide et al., 2018; Moinpour et al., 2020), potentially allowing the formation of different network strengths under otherwise constant polymerization conditions. Previously, weaker hydrogels for dityrosine-crosslinked BSA, I27 and protein L were reported for protein unfolding using 6 M guanidine hydrochloride (Da Silva et al., 2017), while increased entanglements resulted in a higher Young’s modulus for a dityrosine-crosslinked ferredoxin-like globular protein which was previously unfolded with 7 M guanidine hydrochloride (Fu et al., 2021). In contrast to the uncharged chaotropic agent urea, the salt guanidine hydrochloride ionizes in aqueous solutions masking electrostatic interactions (Monera et al., 1994). This indicates, that the used protein and protein unfolding mechanism is crucial towards the resulting mechanical properties of dityrosine-crosslinked hydrogels.

As for the polymerization in SPB, hydrogel discs were stored in their preparation buffer (Figure 2) to exclude external stimuli effects by changes of the environmental conditions (e.g., urea concentration) during storage. Therefore, based on these results, it remained unclear whether an increase in dityrosine crosslinks, entanglements, urea-induced gelation reactions or storage medium characteristics are causing the increasing storage modulus.

### 3.3 Elasticity of bovine serum albumin-based hydrogels

In addition, hydrogel elasticity was evaluated with regard to the loss factor (tan *δ* = G”/G’) determined by a frequency sweep analysis (Figure 2C), whereby a loss factor of 0 corresponds to ideal elastic behavior. Hydrogel elasticity significantly increased upon increasing concentration of urea being present in SPB. Since dityrosine crosslinks are known to contribute to the high elastic properties of structural proteins (Andersen, 1964), this finding may point towards an increase of dityrosine crosslinks being created during hydrogel formation with an increasing urea content. However, no significant change could be observed for the addition of urea at lower concentrations. Several conceivable possibilities could explain this finding, for example protein unfolding altering the number of formed entanglements and/or intermolecular interactions during hydrogel formation or in the formed hydrogel, or the exposure of tyrosine residues being caused by protein unfolding may depend on a threshold concentration of urea. To gain a further understanding of the mechanical properties and the influence of urea, dityrosine quantification by taking advantage of its autofluorescence as reported by Elvin et al. (2005b) and advanced material characterization techniques such as protein structural analysis by circular dichroism spectroscopy or hydrogel structure analysis by small-angle scattering should be conducted prospectively.

### 3.4 Effect of bovine serum albumin concentration on rheological properties

**FIGURE 3:**
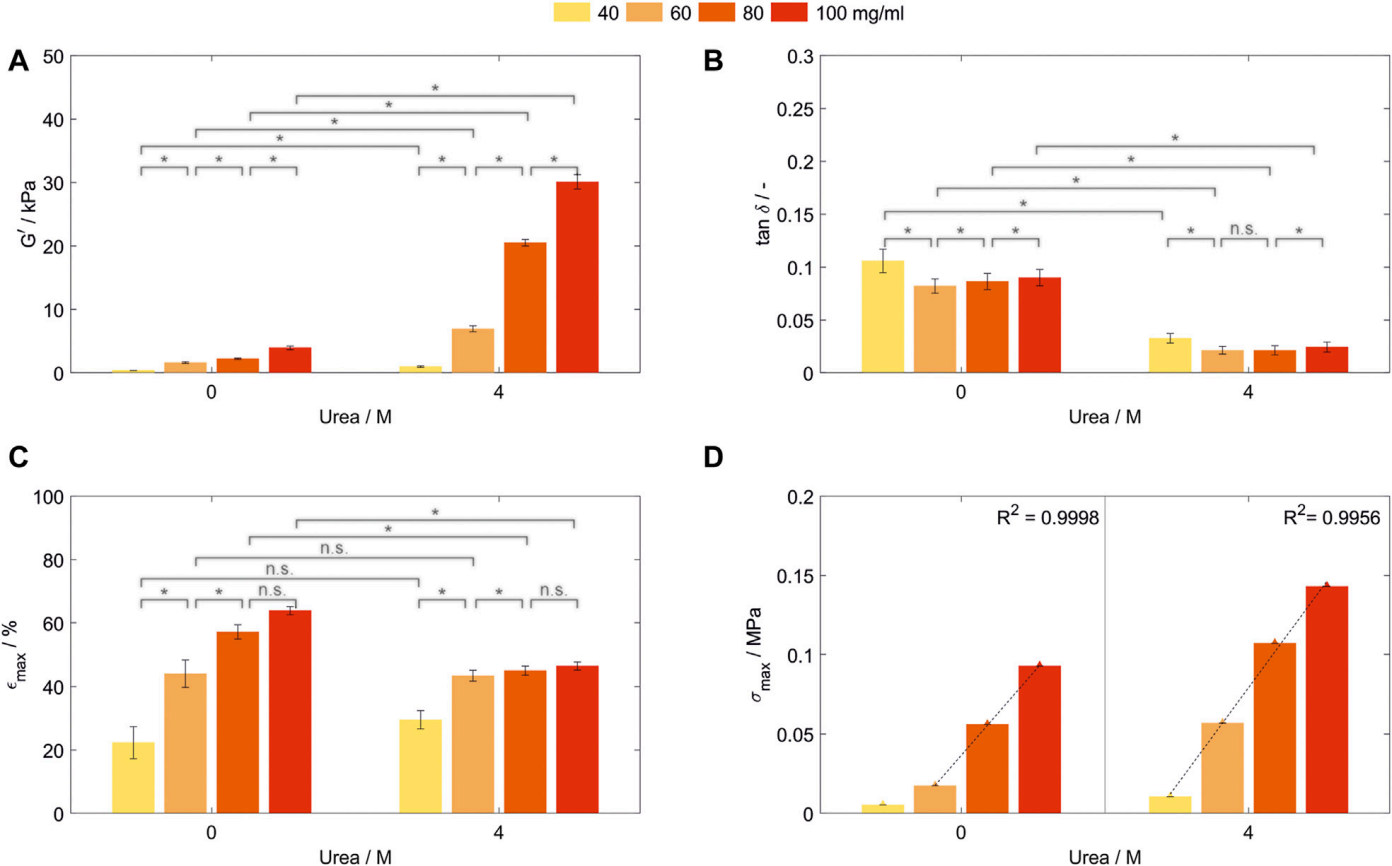
Characterization of hydrogels containing 40, 60, 80, and 100 mg/ml bovine serum albumin prepared in MCB pH 7 with 0 or 4 M urea. All samples were stored in formulation buffer prior analysis. **(A)** Storage modulus (G’) and **(B)** loss factor (tan δ) determined by frequency-dependent oscillatory shear rheology. (*n* = 3) **(C)** Fracture strain (ε_max_) and **(D)** compressive strength (σ_max_) of those hydrogels determined by uniaxial compression tests. Abbreviations: n.s, not significant, *p < 0.05.

In order to investigate the influence of the protein concentration on the resulting mechanical properties, hydrogels with varying BSA concentrations were prepared. Thereby, an optimized buffer system enabled photopolymerization of hydrogels containing between 20 and 100 mg/ml even with 4 M urea being present in MCB pH 7. Hydrogel discs which were stable in their shape could only be obtained for all concentrations above 40 mg/ml. By variation of the BSA and urea concentration in the precursor solution, the storage modulus G’ of the generated hydrogels could be tuned in a range of 0.38 ± 0.02 kPa to 3.93 ± 0.29 kPa (0 M urea) and 0.99 ± 0.14 kPa to 30.05 ± 1.12 kPa (4 M urea) when stored in formulation buffer (Figure 3A).

As for BSA-hydrogels prepared in SPB, for all tested BSA concentrations, G’ (Figure 3A) and elasticity (Figure 3B) were significantly increased when urea was added to the precursor and storage solution. With the exception of the step from 60 to 80 mg/ml prepared without urea (*p* = 0.83, Z = 0.21), an increase in BSA concentration increased G’ significantly (Figure 3A). Interestingly, even though having the lowest network strength, hydrogels containing 40 mg/ml were the least elastic for both urea concentrations tested, while being most elastic for a BSA concentration of 60 mg/ml (Figure 3B). The increase in molecules leads to simultaneous effects such as increasing intermolecular interactions, more surface available and therefore crosslinkable tyrosine residues, more possible chain entanglements—which all are expected to increase the network density—combined with a possibly introduced steric hinderance—all influencing the hydrogel elasticity. Prospective work in material characterization to understand the correlation between these effects has to be conducted to gain a further understanding of the formed hydrogel networks.

### 3.5 Fracture strain and compressive strength of bovine serum albumin-based hydrogels

Fracture strain ε_max_ and compressive strength σ_max_ were determined by uniaxial compression until sample fracture for hydrogels prepared with varying BSA concentrations in MCB pH 7. The stress-strain curve thereby showed an unusual curve shape with multiple drops of the measured stress before increasing again (Supplementary Information S3). During compression, parts of the network are collapsing at lower stresses than the overall hydrogel fracture resulting in this unusual curve shape. However, rheological characteristics showed a high reproducibility of mechanical properties of the intact network structure. Since undirected photopolymerization is used as crosslinking mechanism without aimed crosslinking sites, the reason for the non-reproducible compression curves might be an inhomogeneous network.

The fracture strain increases significantly for both urea concentrations until a concentration of 80 mg/ml BSA without a further significant increase if the concentration is further raised (Figure 3C). Thereby, at least two effects contribute to the fracture strain. Firstly, the increase in protein molecules enables more intermolecular interactions and chemical crosslinks, thus being able to withstand higher stress. Secondly, a large number of these intermolecular interactions and chemical crosslinks reduces the mobility of amino acid chains and concentrates the stress on weak chains within the inhomogeneous network. While the first effect seems to be dominant up to a concentration of 80 mg/ml, the second seems to become more important with increasing protein concentration.

Hydrogels prepared without urea showed a significantly higher maximum strain up to 57.2% ± 2.3%/63.9% ± 1.3% (80/100 mg/ml, 0 M urea) compared to the samples prepared in the presence of urea with 45.0% ± 1.4%/46.5% ± 1.3% for 80/100 mg/ml (Figure 3C). By the addition of urea, no significant change in maximum strain could be observed for 40 mg/ml BSA (*p* = 0.2303). This might be attributed to synergistic effects of an increased crosslinking density and changes in the protein hydrophobicity affecting the surface charge of BSA and thus electrical repulsion between proteins–with the latter being reported as a method for strain-stiffening of protein-based hydrogels (Gu et al., 2022). Since both conditions were stored without external stimuli in their formulation buffer, the influence of the presence of urea in the hydrogel network has to be further investigated.

Interestingly, the compressive strength of the hydrogels shows linear dependency with the protein concentrations for BSA-based hydrogels prepared without urea in the range from 60 to 100 mg/ml (R^2^ = 0.9998) and the whole concentration range tested when urea was present in the precursor solution (R^2^ = 0.9956—Figure 3D). The same trend was observed for the hydrogel toughness (Supplementary Information S4). This linearity could not be seen for any other mechanical property assessed. Further enhancement of the compressive strength and toughness can be achieved by addition of 4 M urea in the precursor solution and storage medium, increasing the compressive strength between 154% from 0.093 to 0.143 MPa (100 mg/ml) and 328% from 0.017 to 0.057 MPa (60 mg/ml) and the toughness between 136% from 4.1 to 5.5 kJ/m³ (100 mg/ml) and 239% from 0.9 to 2.2 kJ/m³ (60 mg/ml). This indicates that the compressive strength and toughness can be modulated in a wide range depending on the specific conditions of hydrogel composition, urea content in the precursor solution and storage conditions.

### 3.6 Influence of preparation and storage buffer

**FIGURE 4:**
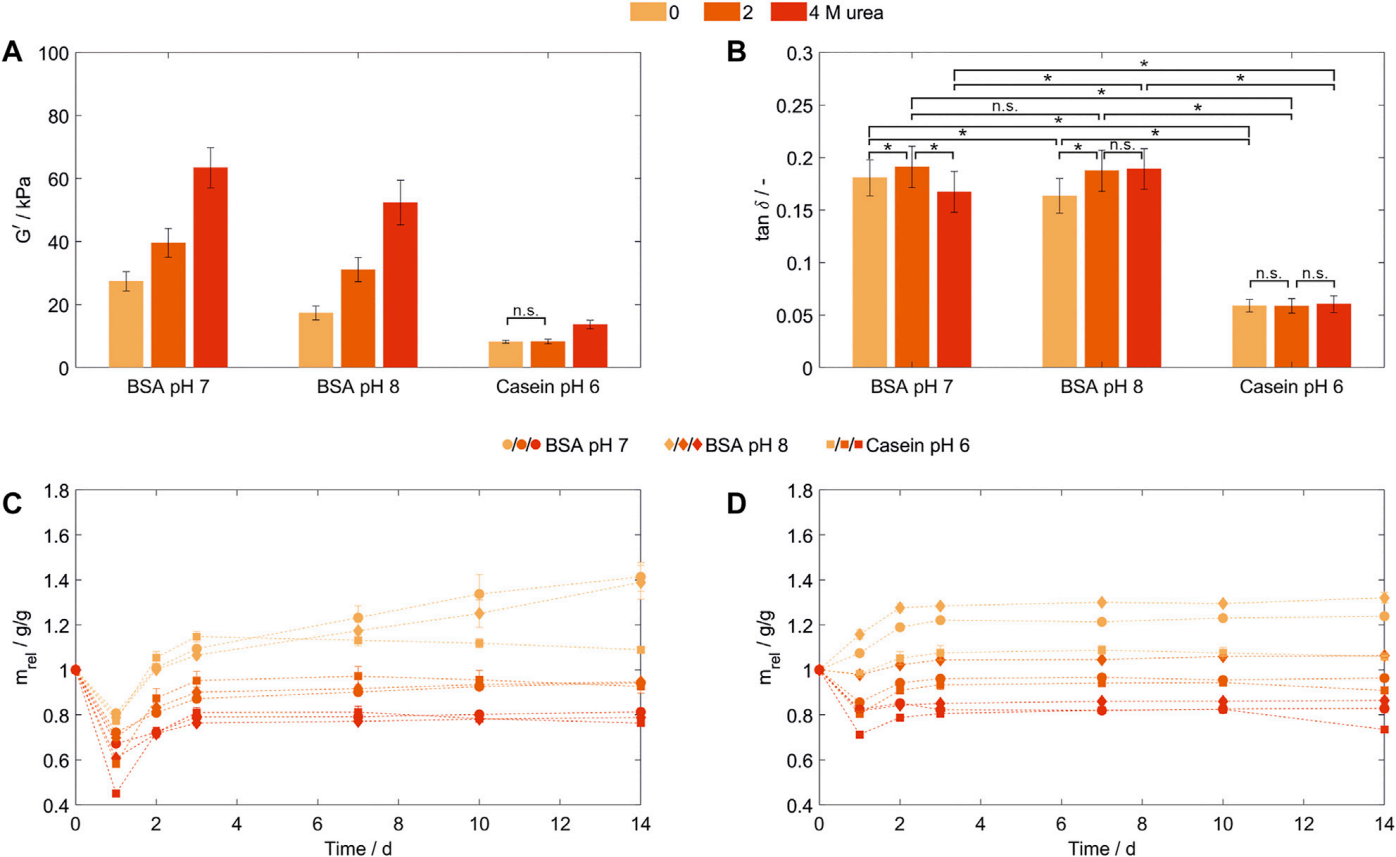
Characterization of hydrogels containing 100 mg/ml bovine serum albumin (pH 7 and 8) or casein (pH 6) prepared in MCB containing 0, 2 or 4 M urea. **(A)** Storage modulus (G’) and **(B)** loss factor (tan δ) as determined by frequency-dependent oscillatory shear rheology. All samples were stored in DPBS (*n* = 3) **(C,D)**: Relative weight swelling ratio (m_rel_) of BSA- and casein-based hydrogels stored in **(C)** DPBS or **(D)** MCB without urea at formulation buffer pH. (*n* = 3).

To exclude effects caused by the presence of urea during storage, a buffer exchange to a dedicated storage buffer prior to storage and analysis was performed following hydrogel formation (Figure 4). DPBS was chosen as a frequently used physiological buffer for sample storage, which exerts a change in environmental conditions on the hydrogels through pH value, type and concentration of ions. For this evaluation, 100 mg/ml BSA- and casein-based hydrogels were produced in MCB containing 0, 2, and 4 M urea. All resulting BSA-based hydrogels showed higher storage moduli in a range from 17.29 ± 2.19 kPa to 63.39 ± 6.42 kPa (Figure 4A) compared to those prepared and stored in SPB (Figure 2A). Increasing urea concentrations in the precursor solution of the BSA-based hydrogels resulted in a significant increase of all storage moduli (302% increase for the formulation buffer pH 8 and 232% for pH 7, respectively, when gels prepared with 4 M urea are compared to urea-free gels). Since the increase in G’ could be observed following a buffer exchange to a similar dedicated storage buffer prior to hydrogel storage, the increasing storage moduli seem not to be related to the different storage medium characteristics discussed before.

### 3.7 Preparation pH of bovine serum albumin-based hydrogels

To vary the intermolecular interactions during hydrogel preparation in the presence and absence of urea, the protein surface charge was altered for hydrogels stored in DPBS. Therefore, BSA-based hydrogels were prepared at two different pH values known not to induce structural transitions to neglect changes in the amount of surface available tyrosines.

A significant higher network density—even in the presence of urea during formulation and hydrogel formation—was achieved when the hydrogels were polymerized closer to their pI, as being previously reported for albumin hydrogels produced by heat-induced denaturation (Park et al., 1998) (Figure 4A). The net surface charge of a protein mainly corresponds to the pH value of protein solutions and increases with an increasing distance to the isoelectric point (pI). Globular BSA with a pI around pH 5.0 to 5.2 (Brown et al., 2004) and a theoretical pI based on its amino acid sequence of pH 5.82 (P02769, UniProtKB/Swiss-Prot), has a net surface charge of −30 (pH 7) and −46 [pH 8, both calculated using Prot pi and ProMoST as pKa database (Josuran, 2022)]. Thus, a lower protein-protein repulsion was expected at pH 7 compared to pH 8 in the formulation buffer and during hydrogel formation explaining the higher network density at pH 7 due to more intermolecular interactions.

For BSA-based hydrogels prepared at pH 7, elasticity was decreased comparing 0 and 2 M urea, while upon addition of 4 M urea a significant increase (*p* = 6·10^−13^, Z = 7.19) compared to 2 M urea could be shown (Figure 4B). In comparison to the preparation at different pH values for gels prepared without urea, elasticity increased significantly at pH 8 (tan *δ* = 0.163 ± 0.017) compared to pH 7 (tan *δ* = 0.181 ± 0.017, *p* = 2·10^−10^, Z = 6.34). As those gel specimens were produced with the same protein concentration, stored in the same buffer system and the dityrosine content was assumed to be similar, changes intermolecular interactions, here induced by formulation buffer pH, showed to significantly influence the hydrogel elasticity and network density.

### 3.8 Casein-based hydrogels

Besides the globular protein BSA, with casein a conjugated protein was introduced for hydrogel formation. Casein consists of four subunits without a stable secondary or tertiary structure (Fox, 2003). In order to fulfill their biological function of calcium and phosphate transportation, these subunits are forming micelles, whose detailed structure and organization are still under investigation (De Kruif et al., 2012). Casein-based hydrogels prepared at pH 7 and 8 showed sample shrinking and macroscale ruptures directly after polymerization (not shown).

Possibly, the chain rearrangements after the introduction of a constrained structure into the typically less structured casein subunits in combination with possible entanglements of chains within these structures lead leads to tensions with the ability to disrupt the entire hydrogel structure, however this was not further assessed. For casein-based hydrogels prepared in MCB at pH 6, these macroscale fractures were not observed and mechanical properties after storage in DPBS were determined. Consistent with the results discussed for BSA, an increasing storage modulus could be observed for increasing urea concentrations in the precursor solution—with the difference that significant changes could only be observed for 4 M urea being present in the precursor solution while elasticity seems to be independent of the urea concentration (Figure 4B). Protein unfolding as discussed for BSA cannot be a suitable explanation since casein subunits do not have a stable secondary or tertiary structure. Though, by the addition of urea micelle integrity will be disrupted, while even at concentrations of 6 M urea, particles in the original micelle size can be traced (De Kruif and Holt, 2003). To evaluate whether the disruption of casein micelles enabled the formation of more dityrosine crosslinks due to an enhanced surface accessibility of tyrosine residues or whether other increased inter- or intramolecular interaction are causing the increased network density, dityrosine quantification and structural analysis of casein and the resulting hydrogels should be conducted prospectively.

### 3.9 Swelling behavior of casein- and bovine serum albumin-based hydrogels

Hydrogel swelling behavior is influenced by the competition between the Donnan osmotic pressure and the elasticity of the hydrogel network (Chang et al., 2011). In general, a swelling medium without added urea was chosen, as the presence of urea is known to lead to a collapse of hydrogel structures (Goh et al., 2017). The latter was confirmed during initial experiments where hydrogels were stored in DPBS (Figure 4C). All hydrogels lost weight after preparation and storage for the first 24 h compared to their initial weight at *t* = 0 to which the liquid volume and buffer substances dissolved in it also contribute in addition to the actual hydrogel network. While hydrogel formation in the absence of urea resulted in a weight loss between 19% (BSA pH 7, 0 M urea) and 23% (Casein pH 6, 0 M urea) of their weight, adding urea enhanced this effect up to a weight decrease of 55% (Casein pH 6, 4 M urea). After the first (*t* = 24 h) and second (*t* = 48 h) buffer exchange, all hydrogels are gaining weight. For hydrogels formulated and prepared without urea, the preparation weight is reached or surpassed at the second day up to a weight increase of 41% (BSA pH 7), 39% (BSA pH 8) and 9% (Casein pH 6) after 14 days. All other conditions do not reach their starting weight in the considered time period. The weight loss after 14 days for 2 M urea is in a range of 5% (BSA pH 8) to 7% (Casein pH 6), while being with 19% (BSA pH 7) to 24% (Casein pH 6) higher for 4 M urea. Thus, the swelling behavior of hydrogels formed in the presence of urea (preparation stage) correlates with the urea concentration used during network formation.

As all hydrogels were stored in a dedicated storage buffer system with a change in ionic strength (from 96 mM salts in the MCB to 150 mM in DPBS), pH (pH 6,7 or 8 in MCB to pH 7 to 7.3 in DPBS) and urea concentration, multiple external stimuli were applied independently or simultaneously. To exclude the influence of changing salt concentrations when altering the pH—normally associated with common buffer systems—the corresponding hydrogel was stored at different pH values was tested in MCB without urea for all conditions (Figure 4D). Thereby, BSA-based hydrogels stored in their formulation buffer directly swelled by 7% (pH 7) to 16% (pH 8), while the casein-based hydrogel still lost 2% of its weight. Hydrogels prepared with urea being present showed a urea concentration-dependent weight loss during storage, high urea concentrations (*c* = 4 M) resulted in a shrinkage of up to 29% for casein and 18% for both BSA-based hydrogels. From day 2 on, all hydrogels showed a swelling behavior, while the swelling degree until the second day was urea-dependent (3%–8% for hydrogels prepared at 4 M urea compared to a swelling degree of 11%–12% for hydrogels prepared without urea).

In summary, four findings concerning the swelling behavior can be highlighted. Firstly, an increasing ionic strength decreases the Donnan osmotic pressure due to more ionic interactions between mobile ions and fixed charges inside the hydrogel network (Chang et al., 2011), resulting in shrinking or lower swelling by pushing liquid out of the hydrogel network as seen for DPBS. Secondly, as for the hydrogels prepared in the presence of urea - which makes up as much as 20% of the initial hydrogel weight -, urea diffuses out of the hydrogel leading to a weight decrease and simultaneously to an increase in hydrophobic interactions in the hydrogel network, explaining the overall shrinking and weight loss of these hydrogels. Thirdly, all hydrogels subject to any external stimulus showed a shrinking behavior following the first buffer exchange and then started to swell again. Thereby, the urge of the hydrogel to swell indicated by an increasing weight is opposed to shrinking effects and weight loss due to the decreasing urea content and/or increasing salt concentration inside the hydrogel network, as discussed before. As for the first day, the shrinking effect seems to be dominant, indicating that either the diffusion of urea out of/salts into the hydrogel network takes more time or the osmotic pressure not being sufficiently due to a too small buffer excess. Fourthly, the protein and formulation buffer pH influence the relative swelling ratio to a certain degree. This may be related to different protein characteristics such as surface charge distribution which is also known to affect swelling ratio in heat-induced BSA-hydrogels (Park et al., 1998). As discussed before, these properties are also responsible for the hydrogel network density requiring further experiments to gain a deeper understanding on the swelling behavior of protein-based hydrogels.

Overall, the protein-based hydrogels presented in this manuscript showed stimuli-responsive swelling behavior. Thereby, hydrogel and buffer characteristics showed to be influencing the relative weight swelling behavior with the more pronounced the differences between swelling medium and formulation buffer, the more shrinking was observed independent of formulation buffer pH or protein.

### 3.10 Predictability of the mechanical properties

The two proteins used in this manuscript show several different characteristics. Just to name some, their amino acid composition, molecular structures and molecular weight differ. For example, 17 disulfide bonds - which are not affected by urea–are stabilizing the molecular structure of the BSA backbone, while there a none in casein. While BSA has a total of 20 tyrosine residues, which corresponds to a tyrosine content of 3.4% (P02769, UniProtKB/Swiss-Prot), the overall tyrosine content in casein is expected to be between 3.9% and 4.2% depending on the exact proportion of its subunits (Kunz and Lönnerdal, 1990; Fox, 2003; Swaisgood, 2003). Due to the loose structure and a higher tyrosine content, a higher surface availability of tyrosine and therefore dityrosine crosslinks content compared to BSA would thus be expected. In contrast, just looking at the adiabatic compressibility, native BSA is with 10.5 10^−11^ Pa^−1^ more compressible than the prevalent casein subunit α-casein with 5.68 10^−11^ Pa^−1^, suggesting more elastic hydrogels being derived from BSA (Gekko and Hasegawa, 1986).

Thus, the resulting mechanical properties of photopolymerized, dityrosine-crosslinked hydrogels derived from proteins are difficult to be foreseen as multiple factors such as the protein characteristics, as well as its folding state and formulation dependent intermolecular interactions during hydrogel formation showed to be crucial process parameters. To compare different proteins with the aim to tune or predict these hydrogel properties, a much deeper understanding of the underlying hydrogel network and its formation will be necessary prospectively.

## 4 Conclusion

BSA and casein were crosslinked through visible light-induced photopolymerization mediated by a ruthenium-based photoinitiator in the presence of urea concentrations up to 4 M. Even though their different protein characteristics, protein-based hydrogels were obtained with significantly different rheological properties, showing the potential of more peptide-constructs or unmodified proteins as prospective material source for hydrogel formation. Depending on the protein, protein concentration, formulation buffer and storage conditions, storage moduli were varied in a range between 0.4 and 63 kDa. The chaotropic agent urea, which is known to weaken hydrophobic interactions, was used as a tool for changing the hydrogel properties. Possibly, urea-induced gelation reactions, or an enhanced surface accessibility of tyrosines or an increasing number of chain entanglements by unfolding BSA and disrupting casein micelles result in higher network densities with simultaneous increase in hydrogel elasticity. As the choice of formulation buffer pH and buffer components are influencing protein characteristics, such as the surface net charge, the individual parameters of the formulation buffer composition as well as the used storage medium have to be chosen carefully in accordance to the desired mechanical properties of polymerized hydrogels.

For the design of bio-based hydrogels or materials for a specific application, characterization of the hydrogel network structure, e.g., by determination of dityrosine content in polymerized hydrogels or small-angle scattering has to be conducted to gain a further understanding of the parameters influencing the mechanical properties. Using this reaction mechanism, naturally occurring, unmodified proteins may be used in future as bio-based materials with potential for biomedical applications, potentially including 3D-printing for the custom design of scaffolds for personalized medicine.

## Data Availability

The raw data supporting the conclusion of this article will be made available by the authors, without undue reservation.

## Glossary

### Abbreviations

3D: three-dimensional
APS: ammonium persulfate
BSA: bovine serum albumin
DAC: dual asymmetric centrifuge
DPBS: Dulbecco’s phosphate-buffered saline
LVR: linear viscoelastic region
MCB: multi-component buffer
pI: isoelectric point
PTFE: Polytetrafluoroethylene
TAPS: N-[Tris(hydroxymethyl)methyl]-3-aminopropanesulfonic acid
MOPSO: 3-Morpholino-2-hydroxypropanesulfonic acid
MWCO: molecular weight cut off
SPB: sodium phosphate buffer

### Greek letters

*α*: significance level of Shapiro-Wilk-Normality test
tan δ: loss factor
ε: engineered strain in %
ε_max_: engineered strain at sample fracture in %
εi,280 nm: molar extinction coefficient of the protein i at 280 nm
σ: engineered stress in Pa
σ_max_: engineered stress at sample fracture in Pa
τ: shear stress in Pa
ω: angular frequency in rad·s^−1^

### Latin letters

A0: original cross section area before compression in m2
F: force in Pa
G’: storage modulus in kPa
G”: loss modulus in kPa
L0: hydrogel length at applied pre-force during compression in m
Lmax: hydrogel length at maximum load during compression in m
m0: hydrogel weight as-prepared
m_rel_: relative weight swelling ratio in g/g
m_t_: hydrogel weight after t days in g
n: number of replicates
R2: coefficient of determination
