# Supplementary material for: Changing mechanical properties of photopolymerized, dityrosine-crosslinked protein-based hydrogels
Source: Front Bioeng Biotechnol. 2022 Sep 12;10:1006438. doi: 10.3389/fbioe.2022.1006438 (PMC9512244; doi:10.3389/fbioe.2022.1006438)
Supplement: Supplementary file 1 [file DataSheet1.docx]

**Supplementary Material**

**Sandra Haas^1^, Saskia Körner^1^, Laura Zintel^1^, Jürgen Hubbuch^*1^**

*^1^ Institute of Engineering in Life Sciences, Section IV: Molecular Separation Engineering, Karlsruhe Institute of Technology (KIT), Fritz-Haber-Weg 2, 76131 Karlsruhe, Germany*

**S1. Hydrogel formulations**

***Table S1 -*** *Summary of hydrogel compositions used to investigate the influence of precursor solution composition and storage conditions on the mechanical properties of dityrosine-crosslinked hydrogels*

| Sample | Precursor solution | | | | | Storage | | | |
| --- | --- | --- | --- | --- | --- | --- | --- | --- | --- |
|  | Protein | Protein concentration | Urea | Buffer | pH | Urea | Buffer | pH | Excess |
| # | - | mg/ml | M | - | - | M | - | - | fold |
| 1 | BSA | 100 | 0 | SPB | 8 | 0 | SPB | 8 | 100 |
| 2 | BSA | 100 | 1 | SPB | 8 | 1 | SPB | 8 | 100 |
| 3 | BSA | 100 | 2 | SPB | 8 | 2 | SPB | 8 | 100 |
| 4 | BSA | 100 | 3 | SPB | 8 | 3 | SPB | 8 | 100 |
| 5 | BSA | 100 | 4 | SPB | 8 | - | - | - | - |
| 6 | BSA | 100 | 0 | MCB | 7 | 0 | DPBS | 7.0 - 7.3 | 25^a)^ |
| 7 | BSA | 100 | 0 | MCB | 7 | 0 | MCB | 7 | 25^a)^ |
| 8 | BSA | 100 | 2 | MCB | 7 | 0 | DPBS | 7.0 - 7.3 | 25^a)^ |
| 9 | BSA | 100 | 2 | MCB | 7 | 0 | MCB | 7 | 25^a)^ |
| 10 | BSA | 100 | 4 | MCB | 7 | 0 | DPBS | 7.0 - 7.3 | 25^a)^ |
| 11 | BSA | 100 | 4 | MCB | 7 | 0 | MCB | 7 | 25^a)^ |
| 12 | BSA | 100 | 0 | MCB | 8 | 0 | DPBS | 7.0 - 7.3 | 25^a)^ |
| 13 | BSA | 100 | 0 | MCB | 8 | 0 | MCB | 8 | 25^a)^ |
| 14 | BSA | 100 | 2 | MCB | 8 | 0 | DPBS | 7.0 - 7.3 | 25^a)^ |
| 15 | BSA | 100 | 2 | MCB | 8 | 0 | MCB | 8 | 25^a)^ |
| 16 | BSA | 100 | 4 | MCB | 8 | 0 | DPBS | 7.0 - 7.3 | 25^a)^ |
| 17 | BSA | 100 | 4 | MCB | 8 | 0 | MCB | 8 | 25^a)^ |
| 18 | Casein | 100 | 0 | MCB | 6 | 0 | DPBS | 7.0 - 7.3 | 25^a)^ |
| 19 | Casein | 100 | 0 | MCB | 6 | 0 | MCB | 6 | 25^a)^ |
| 20 | Casein | 100 | 2 | MCB | 6 | 0 | DPBS | 7.0 - 7.3 | 25^a)^ |
| 21 | Casein | 100 | 2 | MCB | 6 | 0 | MCB | 6 | 25^a)^ |
| 22 | Casein | 100 | 4 | MCB | 6 | 0 | DPBS | 7.0 - 7.3 | 25^a)^ |
| 23 | Casein | 100 | 4 | MCB | 6 | 0 | MCB | 6 | 25^a)^ |
| 24 | BSA | 20 | 0 | MCB | 7 | - | - | - | - |
| 25 | BSA | 40 | 0 | MCB | 7 | 0 | MCB | 7 | 32.5^b)^ |
| 26 | BSA | 60 | 0 | MCB | 7 | 0 | MCB | 7 | 32.5^b)^ |
| 27 | BSA | 80 | 0 | MCB | 7 | 0 | MCB | 7 | 32.5^b)^ |
| 28 | BSA | 100 | 0 | MCB | 7 | 0 | MCB | 7 | 32.5 ^b)^ |
| 29 | BSA | 20 | 4 | MCB | 7 | - | - | - | - |
| 30 | BSA | 40 | 4 | MCB | 7 | 4 | MCB | 7 | 32.5^b)^ |
| 31 | BSA | 60 | 4 | MCB | 7 | 4 | MCB | 7 | 32.5^b)^ |
| 32 | BSA | 80 | 4 | MCB | 7 | 4 | MCB | 7 | 32.5^b)^ |
| 33 | BSA | 100 | 4 | MCB | 7 | 4 | MCB | 7 | 32.5^b)^ |
| 1. *total buffer excess, buffer was exchanged two times (after 24 and 48 hours), total storage time 72 hours* 2. *total buffer excess, buffer was exchanged three times (after 24, 48, and 72 hours), total storage time 96 hours* | | | | | | | | | |

**S2. Linear viscoelastic region of BSA-based hydrogels**

The linear viscoelastic region (LVR) was determined using amplitude sweeps (n = 2) for angular frequencies ω = 1 and 25 s^-1^ and shear stress τ between 5 and 10.000 Pa. In order to avoid inaccuracies due to large deviations in the measured values at low frequencies, the first 10 measured values were averaged to gain an initial value. The shear rate τ corresponding to a decrease (G ') or increase (G' ') by 10% measured from the initial value was determined as the LVR. Besides a significant increase in the storage modulus for 2 and 3 M urea present during polymerization and storage, LVR increased as well for these conditions compared to 0 M urea. Further, no difference between 0 and 1 M urea could be seen. As the LVR is a measure for the ability of the network to withstand shear forces, this indicates an increasing network strength.

***Table S2*** *– Linear viscoelastic region depending on urea content in preparation and storage buffer*

| Urea content  / M | Frequency  / s^-1^ | Linear viscoelastic region  / Pa |
| --- | --- | --- |
| 0 | 1 | 99 ± 23 |
|  | 25 | 142 ± 16 |
| 1 | 1 | 88 ± 10 |
|  | 25 | 90 ± 10 |
| 2 | 1 | 136 ± 15 |
|  | 25 | 282 ± 31 |
| 3 | 1 | 244 ± 5 |
|  | 25 | 324 ± 73 |

**S3. Protein concentration dependent stress-strain curves**

Raw data of the uniaxial compression tests is shown in Figure S2. Comparing the two groups of hydrogels prepared with/without urea, the force increased slower for hydrogels prepared without urea (Figure S2A) and reached higher fracture strain compared to samples prepared with urea (Figure S2B). Multiple cracks before network fracture can be seen in the stress strain curves of hydrogels, here exemplary shown for BSA-based hydrogels prepared without urea with a concentration of 60 mg/ml (Figure S2C) and BSA-based hydrogels prepared with urea with a concentration of 100 mg/ml (Figure S2D). This indicates network inhomogeneities and collapsing of weaker connections before a sudden fracture of the entire hydrogel network.


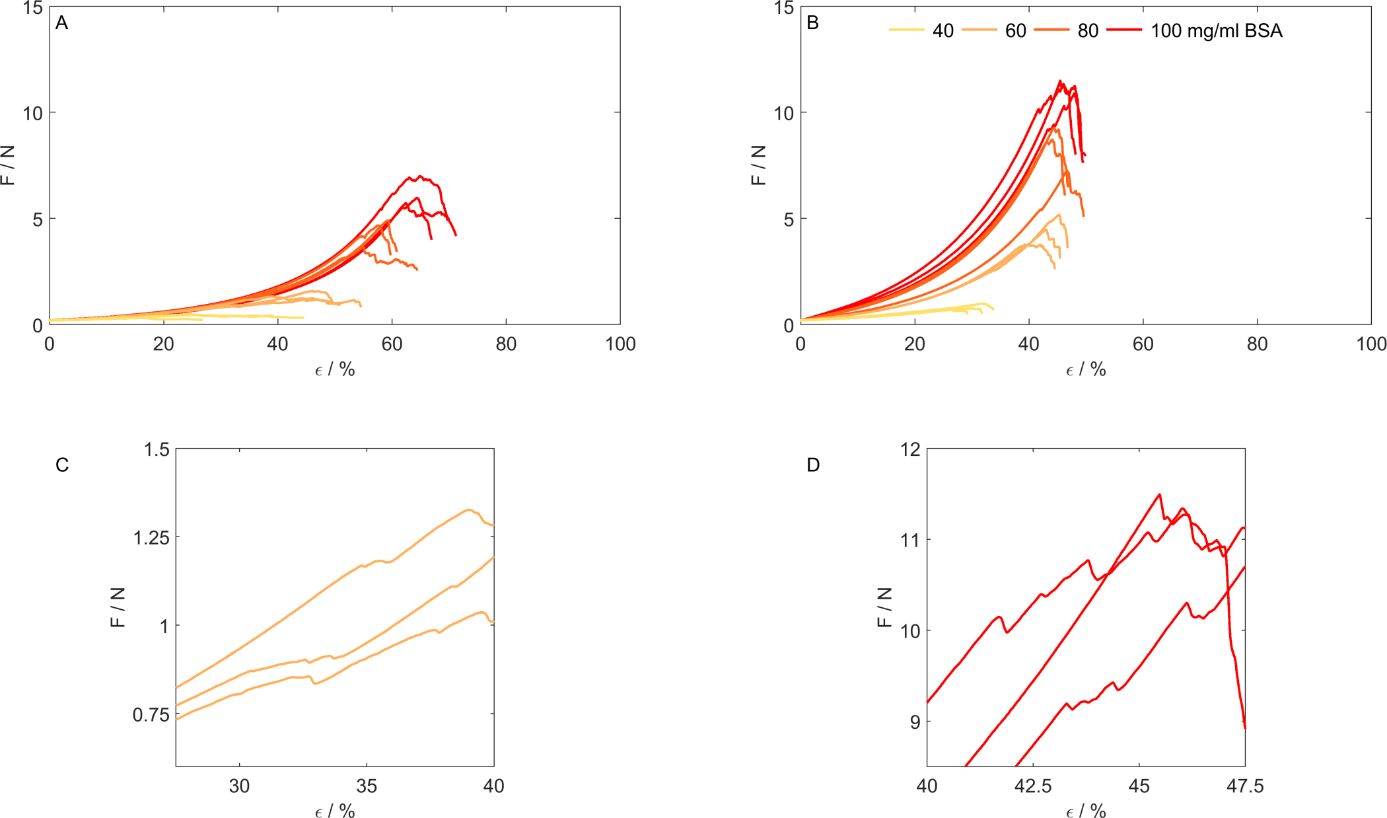


***Figure S1*** *– Stress-strain curves of the uniaxial compression tests performed with BSA-based hydrogels without (A and C) and with (B and D) 4 M urea present during polymerization and storage in a multi-component buffer. A protein concentration of 40 (yellow), 60 (light orange), 80 (orange) and 100 mg/ml (red) BSA was crosslinked. C and D show details of the triplicate measurements: The measured stress showed multiple drops long before sample failure for the two enlarged conditions 60 mg/ml without urea (C) and 100mg/ml with 4 M urea (D). Each line thereby corresponds to one hydrogel sample.*

**S4. Protein concentration dependent hydrogel toughness**

***Table S3*** *– Hydrogel toughness in dependency of the protein concentration for BSA-based hydrogels prepared in MCB without and with 4 M urea in the preparation and storage buffer. Toughness was determined by integrating the stress-strain curves until sample failure and the determination coefficient of a linear fit through 60 – 100 mg/ml (0 M urea) and all data points (4 M urea) is given.*

| Urea content  / M | Protein concentration / mg/ml | Toughness  / kJ/m³ | R² of linear fit  / - |
| --- | --- | --- | --- |
| 0 | 40 | 0.2 ± 0.1 | Not included in the fit |
|  | 60 | 0.9 ± 0.1 | 0.9993 |
|  | 80 | 2.4 ± 0.3 |  |
|  | 100 | 4.1 ± 0.7 |  |
| 4 | 40 | 0.4 ± 0.1 | 0.9998 |
|  | 60 | 2.2 ± 0.3 |  |
|  | 80 | 3.9 ± 0.4 |  |
|  | 100 | 5.6 ± 0.4 |  |
